# Biological Profile and Clinical Features as Determinants for Prolonged Hospitalization in Adult Patients with Measles: A Monocentric Study in Western Romania

**DOI:** 10.3390/pathogens11091018

**Published:** 2022-09-07

**Authors:** Mirela Turaiche, Bogdan Feciche, Adrian Gluhovschi, Felix Bratosin, Iulia Bogdan, Adrian Vasile Bota, Mirela Loredana Grigoras, Camelia Vidita Gurban, Bianca Cerbu, Ana-Olivia Toma, Srivathsava Gurumurthy, Prima Hapsari Wulandari, Iosif Marincu

**Affiliations:** 1 Methodological and Infectious Diseases Research Center, Department of Infectious Diseases, “Victor Babes” University of Medicine and Pharmacy, 300041 Timisoara, Romania; 2 Department of Urology, Satu-Mare County Emergency Hospital, Strada Ravensburg 2, 440192 Satu-Mare, Romania; 3 Department of Obstetrics and Gynecology, “Victor Babes” University of Medicine and Pharmacy Timisoara, Eftimie Murgu Square 2, 300041 Timisoara, Romania; 4 Department of Anatomy and Embryology, “Victor Babes” University of Medicine and Pharmacy Timisoara, Eftimie Murgu Square 2, 300041 Timisoara, Romania; 5 Department of Biochemistry, “Victor Babes” University of Medicine and Pharmacy, Eftimie Murgu Square 2, 300041 Timisoara, Romania; 6 Department of Microbiology, “Victor Babes” University of Medicine and Pharmacy, Eftimie Murgu Square 2, 300041 Timisoara, Romania; 7 Mysore Medical College and Research Institute, Rajiv Gandhi University of Health Sciences, Irwin Road, Mysuru 570001, India; 8 Massachusetts General Hospital, Harvard Medical School, 55 Fruit St., Boston, MA 02114, USA

**Keywords:** measles, infectious disease, public health

## Abstract

Measles is a highly infectious and sometimes deadly illness that is preventable with vaccination. The present research aims to analyze the most recent measles epidemic from Romania that occurred in a population with a falling desire to receive immunizations, by detailing the clinical picture and biological profile of hospitalized patients. A secondary goal of the present research is to identify characteristics that increase the likelihood of a longer hospitalization and the development of measles-related pneumonia. A retrospective cohort study was conducted to follow the course and effects of measles virus infection in adult hospitalized patients who were divided into two groups based on whether they had been in the hospital for more than 6 days or fewer than 6 days. A total of 114 adult patients with measles were eligible to participate in the trial if they had a positive measles-specific IgM antibody test resulting from the study. The average age in the short hospital stay group was 28.1 years, while the average age in the long hospital stay group was 31.9 years. There was a statistically significant difference in the number of Roma persons in the research groups, with 17.4 percent of them having a lengthy hospital stay compared to 5.9 percent in the group with a short hospital stay, according to the findings. It was observed that many patients had a long hospitalization associated with chronic lung disease (OR = 1.07), liver damage (OR = 1.66), Roma ethnicity (OR = 1.79), a long duration elapsed from the last MMR dose (OR = 2.02), elevated c-reactive protein (OR = 2.17), the presence of bilateral pulmonary condensations on X-ray (OR = 3.13), and elevated procalcitonin (OR = 3.49). The same significant independent risk factors were also associated with the development of pneumonia. It is of imperative need to address these risk factors in a patient with measles, moreover in association with an unknown status of vaccination. Vaccination awareness against measles must be pushed in Romania to determine a higher than 95% coverage. Significant efforts are still needed to ensure improved protection against measles epidemics within a specific region or population and, more importantly, in patients with significant risk factors for complications, as described in this study.

## 1. Introduction

Measles is a highly infectious and possibly deadly illness that is preventable with vaccination [1]. It is caused by Measles morbillivirus, generally known as the measles virus. It is placed in the genus Morbillivirus, the family Paramyxoviridae, and the subfamily Orthoparamyxovirinae [2]. The measles virus is an enclosed virus with a diameter of around 100–300 nm and a single-stranded, negative-sense, non-segmented RNA [3]. The predicted annual global mortality toll from measles declined to fewer than 100,000 in 2016, but climbed to more than 200,000 in 2019 [4]. Nonetheless, between 2000 and 2019, the measles vaccine prevented an estimated number of more than 25 million deaths globally [5], while the current SARS-CoV-2 pandemic may have altered the disease’s usual pattern of dissemination and vaccination practices [6,7]. As of July 2022, Romania had recorded a total of 20,204 cases and 64 deaths since the beginning of the ongoing epidemic in 2016 [8].

Humans are the only natural hosts of the measles virus, and only one serotype has been identified, allowing for vaccination-based protection against the illness [9]. Despite the fact that a safe and effective vaccination has been available for almost 60 years, measles continues to cause significant morbidity and death, particularly in resource-poor countries [10]. Measles continues to spread across Europe as a consequence of inadequate vaccine coverage and population immunity gaps, with Eastern Europe, especially Romania, being one of the most impacted areas [11]. Nonetheless, a low-cost, safe vaccination is available, and ongoing worldwide efforts are being made to eradicate the illness. To do this, however, a very high vaccination coverage of at least 95 percent must be acquired and sustained over an extended period of time [12]. However, Romania has an immunization rate below 95% [13].

The measles virus is disseminated by aerosolized droplets and secretions from the respiratory tract [14]. The incubation period, which follows a log-normal distribution, is around 10–14 days and may extend to 23 days, with symptoms lasting up to 3 weeks [15]. The infectious period begins four days before and ends around four days after the commencement of the rash [16]. Measles is extremely infectious, and a single infected case is expected to create on average 12–18 secondary infections in an entirely susceptible community; however, estimates vary greatly depending on the epidemiological situation [17].

Numerous reasons contribute to the unsatisfactory results of measles prevention and control in Romania, including vaccine supply issues, public attitudes about vaccination, and even the lack of a legal framework governing vaccination [18]. Although the disease burden caused by measles had significantly diminished in Romania by the turn of the century, the outbreaks of 2004–2007 and 2011–2013, as well as the one that started in 2016, demand attention [19]. Therefore, the current study plans to analyze the most recent measles epidemic from Romania, in a population with a declining willingness to receive vaccines, by describing the clinical picture and biological profile of hospitalized patients. A secondary objective of the current study is to determine risk factors for a longer hospitalization and the development of measles-related pneumonia.

## 2. Materials and Methods

### 2.1. Research Design and Ethical Considerations

A retrospective cohort study was conducted to track the progression and consequences of measles virus infection in hospitalized adult patients. We analyzed clinical and paraclinical data from patients admitted to Timisoara’s “Victor Babes” Infectious Diseases and Pulmonology Hospital. The data collection period included the time from 1 January 2016 to 1 January 2020. The study population and important characteristics were identified utilizing a population-based administrative database of patients who visited the same clinic’s inpatient setting over the study period. Our comprehensive database included patient medical records that were protected by privacy regulations and collected with the patient’s consent. This information included the patient’s demographics, medical history, and in-hospital treatments. All patients’ baseline characteristics and procedures were documented in the hospital database and in paper patient records examined by qualified doctors involved in the present investigation. We identified a total of 114 cases of measles in our adult group.

The Local Ethics Committee for Scientific Research at the “Victor Babes” Clinical Hospital for Infectious Diseases and Pulmonology in Timisoara operates in accordance with the provisions of Article 167 of Law No. 95/2006, Article 28 of Order 904/2006, and the EU Good Clinical Practice Directives 2005/28/EC, the International Conference on Harmonization of Technical Requirements for Registration of Pharmaceuticals for Human Use (ICH), and the Declaration of Helsinki—Recommendations Guiding Mediation. On 15 December 2021, the present research was authorized with the approval number 12,571. By completing an informed consent form, all research participants consented to participate in this investigation.

### 2.2. Eligibility Criteria and Study Variables

The first eligibility criterion was for patients to be older than 18 years. Patients were also eligible for inclusion if they were admitted to our clinic with clinical symptoms of measles as defined by the Center for Diseases Control (CDC) in 1983 [20], which included fever, maculopapular rash, and any symptom between cough, coryza, or conjunctivitis. To later confirm the measles infection, the measles IgM antibody detection or measles RNA real-time polymerase chain reaction (PCR) was performed according to existing evidence [21,22]. Patients were excluded from the study if their personal records were incomplete, or the patient consent was refused for involvement in medical or analytical studies. The cases that matched inclusion criteria were distributed in two groups based on the median duration of hospitalization that was 6 days. It is conventionally considered that a hospitalization of more than five days is long [23]; therefore, the group of patients hospitalized for fewer than six days were included in the short stay group, and those staying more than six days comprised the long stay group.

The variables considered for statistical analysis comprised the following: the month when infection happened, patient age, gender, pregnancy status, place of origin (urban, rural), ethnicity (Romanian, Roma), measles vaccination status (unvaccinated, incomplete vaccination, complete vaccination), years from last MMR dose, comorbidities (none, diabetes mellitus, cardiovascular disease, chronic lung disease, others), infection source (family, collective, isolated), complications (upper respiratory tract infection, lower respiratory tract infection, sepsis, acute respiratory failure), signs and symptoms (Koplik’s spots, maculopapular rash, hyperpigmented rash, coryza, fever, cough, headache, diarrhea, fatigue, altered mental status), chest X-ray (bilateral consolidation, interstitial pattern), antibiotic treatment (none, cephalosporins, fluoroquinolones, macrolide, penicillins), length of hospital stay, ICU admission, laboratory parameters (white blood cells, lymphocytes, red blood cells, hemoglobin, alanine aminotransferase, aspartate aminotransferase, blood urea nitrogen, creatinine, lactate dehydrogenase, procalcitonin, c-reactive protein, fibrinogen), and infection outcome (mortality).

### 2.3. Statistical Analysis

Data were statistically analyzed using IBM SPSS v.26 (IBM Corp., Chicago, IL, USA) and MedCalc v.20 (MedCalc Software Ltd., Ostend, Belgium). We estimated the absolute (n) and relative (percent) frequencies of categorical variables and used Chi-square and Fisher’s exact tests to compare their proportions. The Mann–Whitney test was used to compare non-Gaussian variables with median and interquartile range definitions (IQR). The Student’s *t*-test was utilized to compare the mean and standard deviation of continuous data having a normal distribution (unpaired, independent samples). Finally, after adjusting for confounding variables, a multivariate analysis was utilized to identify independent risk factors for longer hospital stay in patients with measles. The alpha value was set at 0.05 as a threshold of significance.

## 3. Results

**Figure 1:**
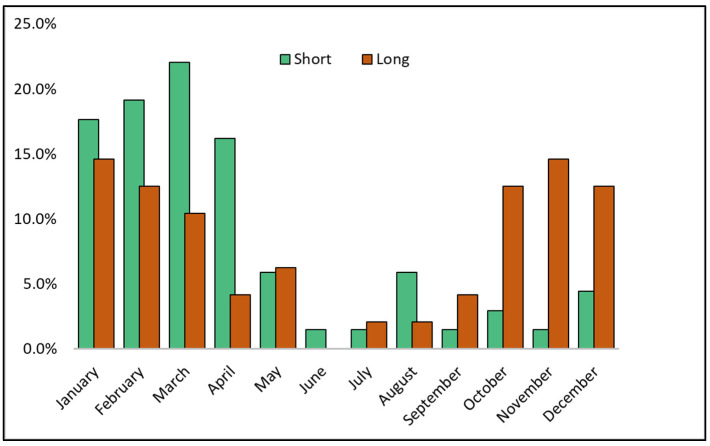
Comparison of measles cases’ proportions by month of infection with stratification by length of hospital stay.

After data collection and patient exclusion based on the study protocol and differential diagnosis, a total of 114 adult patients with measles were included for data analysis. A further processing was implemented by stratifying data into two comparison groups based on the duration of hospital admission. There were 68 (59.6%) patients whose hospital admission was shorter than or equal with the median of 6 days in the existing cohort. The remaining 46 (40.4%) patients had a hospital stay longer than 6 days. The average age in the short hospital stay group was 28.1 years old, compared with 31.9 years old in the long stay group, with a statistically significant difference between means (*p*-value = 0.009). The epidemiological analysis of measles infections distributed by month of diagnosis, presented in Figure 1, identified a relative higher proportion of long hospitalization during the October–December period, compared to shorter hospitalizations from January to April. However, the difference was not statistically significant.

### 3.1. Background Analysis

The background characteristics of adult patients admitted to hospital with measles, presented in Table 1, determined a statistically significant difference in the proportion of Roma people in the study groups, where 17.4% of them had a long hospitalization, compared with 5.9% in the short hospital stay group (*p*-value = 0.049). Patients who were admitted to hospital for more than 6 days had an average of 14.8 years elapsed since their last MMR dose, compared to 6.6 years in patients from the short hospitalization group (*p*-value < 0.001). There were significantly more patients unvaccinated or with a status of incomplete measles vaccination in the group of patients who had a long hospital stay (5.9% incomplete vaccinations vs. 21.7% incomplete vaccinations in the long stay group, *p*-value = 0.036). It was also observed that proportions of comorbidities were significantly different between the two study groups. Thus, diabetes mellitus and chronic lung disease were more prevalent in the long hospitalization group (6.5% vs. 0.0%, *p*-value = 0.033). Additionally, 94.1% of adult patients with measles did not have any comorbid conditions, compared with 73.9% in the long hospitalization group (*p*-value = 0.002).

### 3.2. Clinical Profile and Biological Parameters

**Figure 2:**
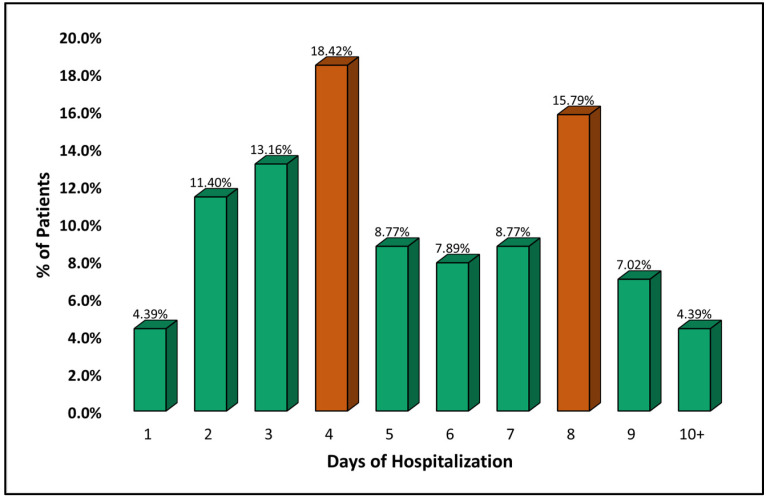
Distribution of patients by duration of hospitalization.

Table 2 describes the clinical characteristics, complications, and outcomes of adult patients admitted with measles, stratified by duration of hospital stay, as described in Figure 2. The most prevalent signs and symptoms were fever (92.6% in the short stay group, and 100% in the long stay group), maculopapular rash (96.8% in the short stay group, and 84.8% in the long stay group), followed by cough that affected more than 80% of all patients. Significant differences were observed in the prevalence of diarrhea (*p*-value = 0.007), fatigue (*p*-value = 0.003), and altered mental status (*p*-value = 0.045), with the highest proportion among patients with a long hospitalization. The same group of patients suffered complications such as liver injury and pneumonia in higher numbers than patients with a shorter hospitalization (28.3% vs. 13.2%, *p*-value = 0.046) and (52.2% vs. 32.4%, *p*-value = 0.034), respectively. The chest X-ray revealed statistically more patterns of bilateral consolidation and interstitial inflammation in patients with longer hospitalization. A total of 39 (89.1%) of these patients received antibiotics, compared to 40 (58.8%) among those who stayed fewer than 7 days in the hospital. The most commonly used antibiotic was cephalosporins in approximately 50% of all treated patients. Lastly, ICU admissions were significantly more frequent in patients with long hospitalization, compared with the other group (10.9% vs. 1.5%, *p*-value = 0.027), although there were no deaths reported in the study cohort.

The biological profile of a complete blood count, and liver, kidney, and inflammatory serum markers of adult patients with measles is presented in Table 3. It was observed that a significantly higher proportion of patients who stayed in hospital more than 6 days had their serum parameters outside the normal range, compared to those with a short hospitalization. Therefore, the white blood cell count, number of lymphocytes, alanine aminotransferase, lactate dehydrogenase, procalcitonin, c-reactive protein, and fibrinogen were, statistically, significantly more elevated. Conversely, hemoglobin was lower in patients with long hospitalization (41.2% vs. 22.1%, *p*-value < 0.001).

### 3.3. Risk Factor Analysis

**Figure 3:**
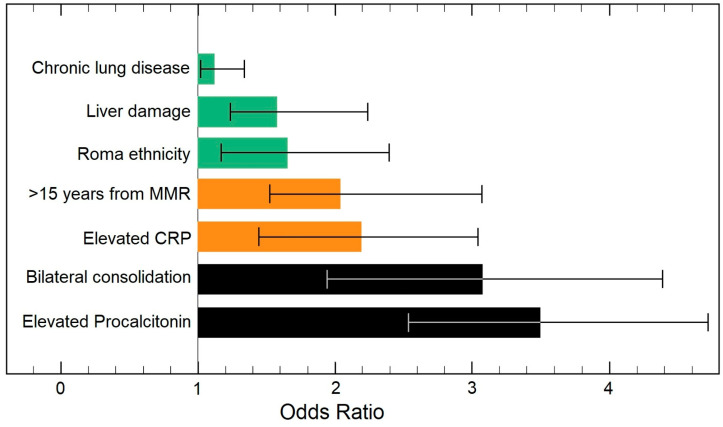
Multivariate risk factor analysis for long hospitalization in adult patients with measles.

**Figure 4:**
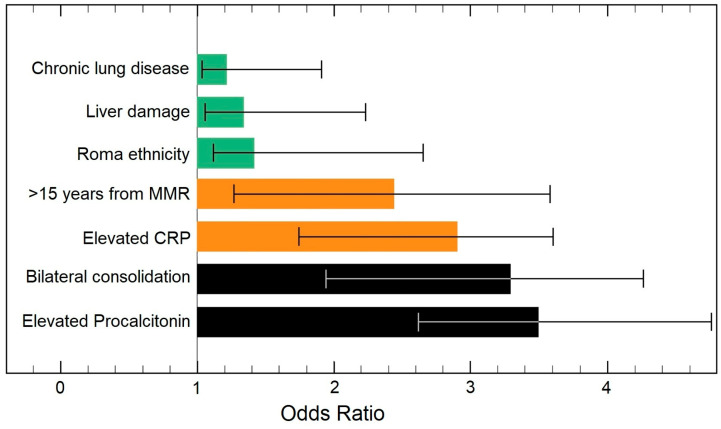
Multivariate risk factor analysis for pneumonia in adult patients with measles.

The risk factor analysis presented in Table 4 and Figure 3 and Figure 4 identified, in ascending order of odds ratios, chronic lung disease, liver damage, Roma ethnicity, duration from the last MMR dose, CRP, bilateral pulmonary condensation on X-ray, and elevated procalcitonin as statistically significant independent risk factors for long hospitalization and the development of pneumonia in adult patients with measles. The analysis was adjusted for confounding factors such as measles positive vaccination status.

## 4. Discussion

The current study presented in detail the biological profile and clinical features as determinants for prolonged hospitalization in adult patients with measles. It was observed that many patients had a long hospitalization associated with chronic lung disease, liver damage, Roma ethnicity, a long duration elapsed from the last MMR dose, elevated c-reactive protein, the presence of bilateral pulmonary condensations on X-ray, and elevated procalcitonin.

### 4.1. Literature Findings and Treatment Options

It was observed among our patients that few were previously vaccinated for measles, or some of them did not have a complete record for measles vaccination. Moreover, almost one third of patients who had a long hospitalization were unvaccinated or had an incomplete vaccination status. Moreover, it was observed in this group of patients that significantly more of them presented with an abnormal laboratory profile, such as elevated procalcitonin, that can occur, for example, due to the presence of a secondary infection such as pneumonia. Roma ethnicity was found to be an important risk factor for longer hospitalization, likely due to the very low vaccination rates in this community in Romania. Similar findings were described in 2020 in a measles outbreak in a Roman community from Slovakia [24]. Another risk factor identified in this study is the liver injury that often occurs in unvaccinated patients and with higher severity [25].

Although vaccination was proven to be extremely effective in preventing infection with the measles virus, a small number of patients will experience symptoms or even a maculopapular rash if there is an immune deficiency, or in cases where the vaccine was administered a very long time ago [26]. Additionally, the measles vaccine’s efficacy is dropping since recent trends show a hesitancy in receiving vaccines, moreover after the vaccination crisis during the COVID-19 pandemic when the general trust in vaccines decreased significantly [27,28]. Research studying the immunogenicity in the long term after measles vaccination versus natural infection in 611 patients, observed that the proportion of individuals with non-detectable protective anti-measles IgG was higher among those who were fully vaccinated than among people who reported having had measles in the past [29]. We encountered several cases of measles in pregnant women, although their evolution was favorable and without significant severe complications. Other studies describe that the measles virus has been shown to infect the placenta, with viral components being found in the syncytiotrophoblast, but no virus-related malformation has been reported [30]. The rate of malformations reported in pregnant measles patients is equivalent to the rate recorded in the general population [31]. While transfer of infection to the fetus has never been verified, placental injury has been recorded, but the specific mechanism by which it happens is unknown, as alteration of the placenta explains its malfunction and, therefore, fetal demise. Transmission to the fetus occurs solely during the labor or delivery period [32]. There is no effective medication to prevent neonatal transmission, although there is a case for immunoglobulin prophylaxis, and the only effective weapon is prevention, therefore, vaccination must happen before pregnancy [33].

Although no effective curative treatment protocol exists for measles and only supportive medication is used, it is described in the literature that several antivirals are often used alone or in combination. Ribavirin and interferon are two examples of this. Indeed, the literature on treatment is scant, and the same indications are mostly based on clinical examples, where the majority of them support the use of ribavirin, particularly at higher dosages [34]. These findings indicate that ribavirin has a favorable impact on measles and that every measles patient, regardless of length of illness, should receive ribavirin in addition to normal symptomatic management. The dose is not well established, although repeated 200,000 IU seems to have therapeutic benefits. Notably, there is no unanimity on the optimal dose of ribavirin. Patients were treated with 50 mg/kg/day at a dose of 1 g every 6 h, with or without loading doses [35]. In this manner, an Indian randomized clinical trial performed on a small sample of 50 patients observed that the length and intensity of fever, constitutional symptoms and maculopapular rash were significantly decreased in the group of patients treated with ribavirin, without having any reported measles complications compared with the control group, where the duration of hospital stay was also significantly longer [36]. The use of steroids is also examined, as their efficacy in some instances of measles pneumonia is recorded. However, our patients did not benefit from such medication.

Additional treatment options include the injection of immunoglobulins, particularly those specific for measles [37], but it is anticipated that the amount of antibodies in plasma pools from donors would decrease as the number of vaccinated donors grows and as the spread of the wild-type measles virus is reduced by herd immunity [38]. Even though the patients included in the current study received only supportive medication and antibiotics when indicated, various clinical data support the use of vitamin A since it is thought to promote epithelial cell turnover, particularly in the respiratory and intestinal tracts [39]. Vitamin A has a greater impact on children under the age of two years, while anecdotal evidence suggests that it also has beneficial effect on adults as well [10].

### 4.2. Study Limitations and Future Perspectives

The current study brings together important information regarding the epidemiology of measles in Romania, and the clinical and paraclinical manifestations in the unvaccinated adult population, as well as about the determinants of longer hospitalization. However, several limitations should be mentioned. Firstly, the sample size was relatively small, which affects the power of the statistical analysis. However, the sample was limited by our hospital database and by the rarity of measles in a country where the vaccination campaign was seriously and successfully implemented during the past 30 years. Secondly, the retrospective design of the study relies on the accurate recordkeeping of patient information, as well as on the accuracy of data being transcribed digitally from paper records.

## 5. Conclusions

In light of the findings in this study indicating a decreasing trend in vaccination against measles in Romania, significant efforts are still required to ensure improved protection against measles epidemics within a specific region and, more importantly, in patients with significant risk factors for complications, as described in this study. It was found in the studied population that chronic lung disease, liver damage, Roma ethnicity, duration from the last MMR dose, CRP, bilateral pulmonary condensation on X-ray, and elevated procalcitonin were independent risk factors for long hospitalization and the development of pneumonia.

We must also consider the economic savings associated with measles prevention on a national basis since prolonged hospital stays are expensive and require isolation and the use of hospital beds that could be allocated for other categories such COVID-19 patients. Catch-up vaccinations with up to two doses of the MMR vaccine continue to be recommended for all individuals who do not know their measles vaccination status, particularly older people who did not benefit from vaccination campaigns, in order to achieve a country-wide vaccination coverage of more than 95%. This research on measles might be used for other illnesses such as COVID-19, therefore preventing deaths and cost savings. Finally, eradicating measles in Romania, as in more wealthy nations such as the United States, would need concerted efforts on a large scale.

## Figures and Tables

**Table 1:** Comparison of background characteristics of adult patients admitted with measles by duration of hospital stay.

Variables	Short Stay (*n* = 68)	Long Stay (*n* = 46)	*p*-Value *
**Background**			
Age (mean ± SD)	28.1 ± 7.0	31.9 ± 8.3	0.009
Gender—female	31 (45.6%)	22 (47.8%)	0.814
Pregnant—yes	5 (7.4%)	1 (2.2%)	0.224
Place of origin—urban	39 (57.4%)	23 (50.0%)	0.439
Ethnicity—Roma	8 (5.9%)	4 (17.4%)	0.049
**Infection source**			0.813
Family	9 (13.2%)	6 (13.0%)	
Collective	3 (4.4%)	1 (2.2%)	
Isolated case	56 (82.4%)	39 (84.8%)	
**Vaccination status**			0.036
Unvaccinated	61 (89.7%)	35 (76.1%)	
Incomplete	4 (5.9%)	10 (21.7%)	
Complete	1 (4.4%)	1 (2.2%)	
Years from last MMR dose (mean ± SD)	6.6 ± 4.1	14.8 ± 6.3	<0.001
**Comorbidities**			
None	64 (94.1%)	34 (73.9%)	0.002
Diabetes Mellitus	0 (0.0%)	3 (6.5%)	0.033
Cardiovascular disease	1 (1.5%)	1 (2.2%)	0.779
Chronic lung disease	0 (0.0%)	3 (6.5%)	0.033
Others	3 (4.4%)	5 (10.9%)	0.185

* Data reported as n (%) and analyzed using Chi-square or Fisher’s exact test. Data stratified by duration of hospital admission as short stay (less than or equal with the median of 6 days) and long stay (more than the median of 6 days); SD—standard deviation; MMR—measles mumps rubella vaccine.

**Table 2:** Comparison of clinical characteristics, complications, and outcomes of adult patients admitted with measles, stratified by duration of hospital stay.

Variables	Short Stay (*n* = 68)	Long Stay (*n* = 46)	*p*-Value *
**Signs and Symptoms**			
Koplik’s spots	21 (30.9%)	15 (32.6%)	0.845
Maculopapular rash	59 (86.8%)	39 (84.8%)	0.765
Fever	63 (92.6%)	46 (100.0%)	0.059
Coryza	40 (58.8%)	28 (60.9%)	0.827
Conjunctivitis	31 (45.6%)	26 (56.5%)	0.252
Cough	53 (77.9%)	41 (89.1%)	0.123
Headache	29 (42.6%)	17 (37.0%)	0.543
Diarrhea	15 (22.1%)	21 (45.7%)	0.007
Fatigue	42 (61.8%)	40 (87.0%)	0.003
Altered mental status	3 (4.4%)	7 (15.2%)	0.045
**Complications**			
None	6 (8.8%)	0 (0.0%)	0.038
Liver damage	9 (13.2%)	13 (28.3%)	0.046
Upper respiratory tract infection	29 (42.6%)	14 (30.4%)	0.186
Pneumonia	22 (32.4%)	24 (52.2%)	0.034
Sepsis	1 (12.2%)	3 (6.5%)	0.150
Acute respiratory failure	1 (12.2%)	2 (4.3%)	0.346
**Chest X-ray**			
Bilateral consolidation	20 (29.4%)	26 (56.5%)	0.003
Interstitial pattern	18 (26.5%)	20 (43.5%)	0.058
**Antibiotic treatment**			
None	28 (41.2%)	5 (10.9%)	<0.001
Cephalosporins	34 (50.0%)	22 (47.8%)	0.819
Fluoroquinolones	2 (2.9%)	4 (8.7%)	0.177
Macrolide	2 (2.9%)	9 (19.6%)	0.003
Penicillins	2 (2.9%)	6 (13.0%)	0.038
Hospital stay—days (mean ± SD)	4.4 ± 1.2	8.7 ± 1.9	0.001
ICU admission	1 (1.5%)	5 (10.9%)	0.027
**Mortality**	0 (0.0%)	0 (0.0%)	-

* Data reported as n (%) and analyzed using Chi-square or Fisher’s exact test. Data stratified by duration of hospital admission as short stay (less than or equal with the median of 6 days) and long stay (more than the median of 6 days); IQR—interquartile range; ICU—intensive care unit.

**Table 3:** Comparison of biological parameters of adult patients admitted with measles, stratified by duration of hospital stay.

Variables	Normal Range	Short Stay (*n* = 68)	Long Stay (*n* = 46)	*p*-Value *
WBC (thousands/mm^3^)	4.5–11.0	30.5%	61.5%	<0.001
Lymphocytes (thousands/mm^3^)	1.0–4.8	42.2%	54.9%	0.001
RBC (millions/mm^3^)	4.35–5.65	31.8%	38.0%	0.218
Hemoglobin (g/dL)	13.0–17.0	22.1%	41.2%	<0.001
Platelets (thousands/mm^3^)	150–450	49.7%	60.9%	0.055
ALT (U/L)	7–35	34.9%	47.1%	0.044
AST (U/L)	10–40	38.3%	46.3%	0.192
BUN (mmol/L)	2.1–8.5	22.8%	27.5%	0.373
Creatinine (µmol/L)	0.74–1.35	21.6%	26.6%	0.408
LDH (U/L)	140–280	18.0%	30.6%	0.001
Procalcitonin (ug/L)	0–0.5 ug/L	34.7%	53.1%	<0.001
CRP (mg/L)	0–10 mg/L	32.3%	55.9%	<0.001
Fibrinogen (g/L)	2–4 g/L	36.9%	58.0%	<0.001

* Data reported as n (% outside the normal range), and analyzed using Chi-square or Fisher’s exact test. Data stratified by duration of hospital admission as short stay (less than or equal with the median of 6 days) and long stay (more than the median of 6 days); WBC—white blood cells; RBC—red blood cells; AST—aspartate aminotransferase; ALT—alanine aminotransferase; LDH—lactate dehydrogenase; BUN—blood urea nitrogen; CRP—C-reactive protein.

**Table 4:** Multivariate risk factor analysis for long hospitalization and development of pneumonia using data recorded on patient admission. Data presented in ascending order of odds ratios.

Risk Factors	Long Hospitalization (OR 95% CI)	*p*-Value	Pneumonia (OR 95% CI)	*p*-Value
Chronic lung disease	1.07 (1.01–1.46)	0.044	1.22 (1.03–1.94)	0.044
Liver damage	1.66 (1.28–2.41)	0.018	1.35 (1.06–2.27)	0.017
Ethnicity—Roma	1.79 (1.11–2.59)	0.012	1.41 (1.13–2.70)	0.005
Years from last MMR dose	2.02 (1.42–3.18)	0.004	2.43 (1.22–3.66)	<0.001
CRP	2.17 (1.33–3.10)	0.001	2.96 (1.64–3.69)	<0.001
Bilateral consolidation	3.13 (1.91–4.46)	<0.001	3.28 (1.88–4.33)	<0.001
Procalcitonin	3.49 (2.43–4.77)	<0.001	3.75 (2.63–4.81)	<0.001

CRP—C-reactive protein; MMR—measles mumps rubella vaccine.

## Data Availability

Data available on request.

## References

[1] Moussli N., Kabengele E., Jeannot E. (2019). Measles at Work: Status of Measles Vaccination at a Multinational Company. Vaccines.

[2] Siering O., Cattaneo R., Pfaller C.K. (2022). C Proteins: Controllers of Orderly Paramyxovirus Replication and of the Innate Immune Response. Viruses.

[3] Li T., Shen Q.-T. (2021). Insights into Paramyxovirus Nucleocapsids from Diverse Assemblies. Viruses.

[4] Patel M.K., Goodson J.L., Alexander J.P., Kretsinger K., Sodha S.V., Steulet C., Gacic-Dobo M., Rota P.A., McFarland J., Menning L. (2020). Progress toward regional measles elimination—worldwide, 2000–2019. MMWR Morb. Mortal. Wkly. Rep..

[5] Lindstrand A., Cherian T., Chang-Blanc D., Feikin D., O’Brien K.L. (2021). The World of Immunization: Achievements, Challenges, and Strategic Vision for the Next Decade. J. Infect. Dis..

[6] Taheri S.M., Basti M., Tabatabaei S.M., Rajabkhah K. (2021). Measles, mumps, and rubella (MMR) vaccine and COVID-19: A systematic review. Int. J. Mol. Epidemiol. Genet..

[7] Feldman A.G., O’Leary S.T., Isakov L.D. (2021). The risk of resurgence in vaccine preventable infections due to COVID-related gaps in immunization. Clin. Infect. Dis..

[8] National Institute of Public Health Measles Situation in Romania on 17 July 2020. https://www.cnscbt.ro/index.php/informari-saptamanale/rujeola-1/1871-situatia-rujeolei-in-romania-la-data-de-17-07-2020/file.

[9] Ferren M., Horvat B., Mathieu C. (2019). Measles Encephalitis: Towards New Therapeutics. Viruses.

[10] Hübschen J.M., Gouandjika-Vasilache I., Dina J. (2022). Measles. Lancet.

[11] Pițigoi D., Săndulescu O., Crăciun M.D., Drăgănescu A., Jugulete G., Streinu-Cercel A., Vișan A., Rîciu C., Rafila A., Aramă V. (2020). Measles in Romania—Clinical and epidemiological characteristics of hospitalized measles cases during the first three years of the 2016-ongoing epidemic. Virulence.

[12] Plans-Rubió P. (2020). Are the Objectives Proposed by the WHO for Routine Measles Vaccination Coverage and Population Measles Immunity Sufficient to Achieve Measles Elimination from Europe?. Vaccines.

[13] Stanescu M.A., Totan A., Miricescu A., Stefani D. (2019). Constantin Serban, Bogdan Grajdeanu, Ioana Diaconu. Comparative analysis of cases of measles from Romania, Bulgaria and Hungary in the context of the European Union. Rom. J. Med. Pract..

[14] Laksono B.M., De Vries R.D., McQuaid S., Duprex W.P., De Swart R.L. (2016). Measles Virus Host Invasion and Pathogenesis. Viruses.

[15] Coughlin M.M., Beck A.S., Bankamp B., Rota P.A. (2017). Perspective on Global Measles Epidemiology and Control and the Role of Novel Vaccination Strategies. Viruses.

[16] World Health Organization (2018). Manual for the Laboratory-Based Surveillance of Measles, Rubella, and Congenital Rubella Syndrome, 3rd ed. https://www.who.int/publications/m/item/chapter-1-manual-for-the-laboratory-based-surveillance-of-measles-rubella-and-congenital-rubella-syndrome.

[17] Guerra F.M., Bolotin S., Lim G., Heffernan J., Deeks S.L., Li Y., Crowcroft N.S. (2017). The basic reproduction number (R0) of measles: A systematic review. Lancet Infect. Dis..

[18] Dascalu S. (2019). Measles Epidemics in Romania: Lessons for Public Health and Future Policy. Front. Public Health.

[19] Davitoiu A.M., Spatariu L., Plesca D.A., Dimitriu M., Cirstoveanu C.G., Chindris S. (2021). Review of the measles epidemic in children from Central Eastern Europe in the third millennium. Exp. Ther. Med..

[20] Morbidity Mortality Weekly Report (1983). Classification of measles cases and categorization of measles elimination program. Morb. Mortal. Wkly. Rep..

[21] Hübschen J.M., Bork S.M., Brown K.E., Mankertz A., Santibanez S., Ben Mamou M., Mulders M.N., Muller C.P. (2017). Challenges of measles and rubella laboratory diagnostic in the era of elimination. Clin. Microbiol. Infect..

[22] Bogdan I., Citu C., Bratosin F., Malita D., Romosan I., Gurban C.V., Bota A.V., Turaiche M., Bratu M.L., Pilut C.N. (2022). The Impact of Multiplex PCR in Diagnosing and Managing Bacterial Infections in COVID-19 Patients Self-Medicated with Antibiotics. Antibiotics.

[23] Tipton K., Leas B.F., Mull N.K., Siddique S.M., Greysen S.R., Meghan B., Tsou A.Y. (2021). Interventions to Decrease Hospital Length of Stay [Internet] (Technical Brief, No. 40.) Introduction.

[24] Carazo S., Billard M.N., Boutin A., De Serres G. (2020). Effect of age at vaccination on the measles vaccine effectiveness and immunogenicity: Systematic review and meta-analysis. BMC Infect. Dis..

[25] Citu I.M., Citu C., Gorun F., Motoc A., Gorun O.M., Burlea B., Bratosin F., Tudorache E., Margan M.-M., Hosin S. (2022). Determinants of COVID-19 Vaccination Hesitancy among Romanian Pregnant Women. Vaccines.

[26] Da Silva T.M.R., de Sá A.C.M.G.N., Vieira E.W.R., Prates E.J.S., Beinner M.A., Matozinhos F.P. (2021). Number of doses of Measles-Mumps-Rubella vaccine applied in Brazil before and during the COVID-19 pandemic. BMC Infect. Dis..

[27] Bianchi F.P., Mascipinto S., Stefanizzi P., De Nitto S., Germinario C., Tafuri S. (2021). Long-term immunogenicity after measles vaccine vs. wild infection: An Italian retrospective cohort study. Hum. Vaccines Immunother..

[28] Hudečková H., Stašková J., Mikas J., Mečochová A., Staroňová E., Polčičová A., Baška T., Novák M., Malinovská N., Zibolenová J. (2020). Measles Outbreak in a Roma Community in the Eastern Region of Slovakia, May to October 2018. Slov. J. Public Health.

[29] Dinh A., Fleuret V., Hanslik T. (2013). Liver involvement in adults with measles. Int. J. Infect. Dis..

[30] Kobayashi K., Tajima M., Toishi S., Fujimori K., Suzuki Y., Udagawa H. (2005). Fetal growth restriction associated with measles virus infection during pregnancy. J. Perinat. Med..

[31] Misin A., Antonello R.M., Di Bella S., Campisciano G., Zanotta N., Giacobbe D.R., Comar M., Luzzati R. (2020). Measles: An Overview of a Re-Emerging Disease in Children and Immunocompromised Patients. Microorganisms.

[32] Rasmussen S.A., Jamieson D.J. (2015). What Obstetric Health Care Providers Need to Know About Measles and Pregnancy. Obstet. Gynecol..

[33] Simionescu A.A., Streinu-Cercel A., Popescu F.-D., Stanescu A.M.A., Vieru M., Danciu B.M., Miron V.D., Săndulescu O. (2021). Comprehensive Overview of Vaccination during Pregnancy in Europe. J. Pers. Med..

[34] Hosoya M., Shigeta S., Mori S., Tomoda A., Shiraishi S., Miike T., Suzuki H. (2001). High-dose intravenous ribavirin therapy for subacute sclerosing panencephalitis. Antimicrob. Agents Chemother..

[35] Bichon A., Aubry C., Benarous L., Drouet H., Zandotti C., Parola P., Lagier J.-C. (2017). Case report: Ribavirin and vitamin A in a severe case of measles. Medicine.

[36] Pal G. (2011). Effects of ribavirin on measles. J. Indian Med. Assoc..

[37] Young M.K. (2019). The indications and safety of polyvalent immunoglobulin for post-exposure prophylaxis of hepatitis A, rubella and measles. Hum. Vaccines Immunother..

[38] Audet S., Virata-Theimer M.L., Beeler J.A., Scott D.E., Frazier D.J., Mikolajczyk M.G., Eller N., Chen F.M., Yu M.Y. (2006). Measles-virus-neutralizing antibodies in intravenous immu-noglobulins. J. Infect. Dis..

[39] Surman S.L., Penkert R.R., Sealy R.E., Jones B.G., Marion T.N., Vogel P., Hurwitz J.L. (2020). Consequences of Vitamin A Deficiency: Immunoglobulin Dysregulation, Squamous Cell Metaplasia, Infectious Disease, and Death. Int. J. Mol. Sci..

